# Differential DNA methylation of *MSI2* and its correlation with diabetic traits

**DOI:** 10.1371/journal.pone.0177406

**Published:** 2017-05-24

**Authors:** Jae-Pil Jeon, In-Uk Koh, Nak-Hyun Choi, Bong-Jo Kim, Bok-Ghee Han, Suman Lee

**Affiliations:** 1 Center for Biomedical Science, National Research Institute of Health, Cheongju-si, Republic of Korea; 2 Center for Genome Science, National Research Institute of Health, Cheongju-si, Republic of Korea; University of Rochester, UNITED STATES

## Abstract

Differential DNA methylation with hyperglycemia is significantly associated with Type 2 Diabetes (T2D). Longtime extended exposure to high blood glucose levels can affect the epigenetic signatures in all organs. However, the relevance of the differential DNA methylation changes with hyperglycemia in blood with pancreatic islets remains unclear. We investigated differential DNA methylation in relation to glucose homeostasis based on the Oral Glucose Tolerance Test (OGTT) in a population-based cohort. We found a total of 382 differential methylation sites from blood DNA in hyperglycemia and type 2 diabetes subgroups using a longitudinal and cross-sectional approach. Among them, three CpG sites were overlapped; they were mapped to the *MSI2* and *CXXC4* genes. In a DNA methylation replication study done by pyrosequencing (n = 440), the CpG site of *MSI2* were shown to have strong associations with the T2D group (p value = 2.20E-16). The differential methylation of *MSI2* at chr17:55484635 was associated with diabetes-related traits, in particular with insulin sensitivity (*QUICKI*, p value = 2.20E-16) and resistance (HOMA-IR, p value = 1.177E-07). In human pancreatic islets, at the single-base resolution (using whole-genome bisulfite sequencing), the 292 CpG sites in the ±5kb at chr17:55484635 were found to be significantly hypo-methylated in donors with T2D (average decrease = 13.91%, 95% confidence interval (CI) = 4.18~ 17.06) as compared to controls, and methylation patterns differed by sex (-9.57%, CI = -16.76~ -6.89) and age (0.12%, CI = -11.17~ 3.77). Differential methylation of the *MSI2* gene (chr17:55484635) in blood and islet cells is strongly related to hyperglycemia. Our findings suggest that epigenetic perturbation on the target site of *MSI2* gene in circulating blood and pancreatic islets should represent or affect hyperglycemia.

## Introduction

Epigenetics is the main mechanism that mediates the effects of dietary and lifestyle factors [1]. Lifestyle factors such as diet, tobacco smoking, alcohol consumption, and exposure to environmental pollutants can modify epigenetic patterns [2–5]. Glucose levels are maintained by pancreatic hormones to maintain blood glucose homeostasis within a normal range. Glucose levels increase and decrease in healthy people; however, this may not be the case in people with type 2 diabetes (T2D). Postprandial hyperglycemia is known to be one of the earliest signs of abnormal glucose homeostasis associated with T2D [6]. The rise and fall of postprandial glucose levels is mediated by the first-phase insulin response following food intake. Epigenetic perturbations associated with changes in glucose tolerance over the course of a lifetime can be both a cause and consequence of T2D.

Epigenetic influences on DNA methylation and gene expression are crucial in susceptibility to hyperglycemic diseases, T2D, and obesity [7–10]. A recent epigenome-wide association study (EWAS) showed that differential DNA methylation is significantly associated with T2D and obesity in blood and other tissues [4, 9, 11–13]. Significant differences in DNA methylation profiles related to T2D were found in blood and target tissues, including pancreatic islets. So far, the association of DNA methylation with type 2 diabetes and insulin has been investigated in human pancreatic islets [14, 15], human adipose tissue [16, 17], CD4+ T cells [18], and peripheral blood [13]. The DNA methylation of the hypoxia-inducible transcription factor 3A (*HIF3A*) gene in blood cells and adipose tissue is associated with body mass index (BMI) [4].

We investigated differential DNA methylation related to hypoglycemia based on the Oral Glucose Tolerance Test (OGTT) in a longitudinal population-based cohort. To identify hyperglycemia-related differentially methylated positions (DMPs) using the OGTT, we investigated individuals with high glucose responses (highly elevated glucose level in blood at 60 min, ΔhiGlu60) and type 2 diabetes at different phases in longitudinal and cross-sectional analyses. Candidate DMP in the *MSI2* (Musashi RNA-Binding Protein 2) gene was selected for the replication study in blood and islet cells. In this study, we found that differential methylation of the *MSI2* gene in blood was strongly correlated with glycemic traits, and differential methylation was also found in pancreatic islets from donors with type 2 diabetes. Our study may provide a foundation for future studies exploring this key epigenetic modification in target cells related to glucose homeostasis.

## Materials and methods

### Study subjects

All subjects were recruited from the Korean Genome Epidemiology Study (KoGES), a longitudinal community-based prospective study [19]. All samples from the National Biobank of Korea were obtained with written informed consent, and this study received the Korea National Institute of Health (KNIH) institutional review board (IRB) approval (Trial registration: KNIH 2014-08EXP-05-P-A. Registered 8 August 2014).

Blood samples were obtained based on an oral glucose tolerance test (OGTT) from 26 individuals at two time points separated by 10 years, in the first phase (2001) and in the follow-up 5th phase (2011) of the KoGES (Table 1). For the OGTT assay, the subjects were given 75 g glucose dissolved in 300 ml water (Glucola; Allegiance Healthcare, McGaw Park, IL) to drink within a period of 5 min. Blood samples were obtained at 0, 1, and 2 h after glucose ingestion [6, 20]. Homeostasis Model Assessment (HOMA) was used to estimate insulin resistance (HOMA-IR: Ins0 (μU/mL) × Glu0 (mg/mL)/405). The quantitative insulin sensitivity check index (*QUICKI*) was used to assess insulin sensitivity (*QUICKI*: 1 / (log(Ins0 μU/mL) + log(Glu0 mg/dL).

**Table 1:** Summary of the subjects in the discovery study based on OGTT.

Subgroup		Controls	Cases	p values
**ΔhiGlu60**	**n (male/female)**	8 (4/4)	8 (4/4)	
	**Age (yr)**	51±2.24	52.5±4.85	
	**Glu0**	84.38±14.38	90.25±5.43	0.3290
	**Glu60**	101.88±7.83	203.5±11.73	**< 0.0001**
	**Glu120**	109.875±26.36	170.62±24.60	**0.0005**
**type 2 diabetes**	**n (male/female)**	5 (5/0)	5 (5/0)	
	**Age**	49±2.68	49±2.68	
	**Glu0**	91.4±5.68	168±49.7	**0.0155**
	**Glu60**	143±20.23	344.4±76.14	**0.0009**
	**Glu120**	135.2±39.45	316.2±48.70	**0.0004**

The values are indicated by mean±SD, Glu0 = Fasting Blood glucose level(mg/dL), Glu60 = Blood glucose level at 60 min for OGTT(mg/dL), Glu120 = Blood glucose level at 120 min for OGTT(mg/dL), OGTT = Oral Glucose Tolerance Test

In the discovery set (n = 26), two hyperglycemia subgroups in the follow-up 5th phase were included (Table 1): a high glucose response subgroup (ΔhiGlu60) (cases/controls: 8/8) and a type 2 diabetes subgroup (cases/controls: 5/5). The ΔhiGlu60 subgroup compared individuals with postprandial low versus high glycemia, in which the delta Glu60-Glu0 (difference in glucose levels before and 1 h after OGTT challenge) was lower than 20 or higher than 100, respectively. A diagnosis of diabetes was based on the subject’s health condition at the 5th phase of the KoGES follow-up. Two subgroups were age- and sex-matched with the respective comparison groups. All participants were normoglycemic in the first phase. Subjects with a previous history of diabetes or any malignant disease were excluded.

For the replication set, DNA samples were derived from the blood of study subjects who participated in the 5th phase of the KoGES. The T2D replication group compared non-diabetic subjects (n = 220) versus diabetic subjects (n = 220) who displayed both >126mg/ml fasting glucose level and >200mg/ml Glu120. Subjects with a previous history of diabetes or any malignant disease were excluded.

For whole genome bisulfite sequence (WGBS) of pancreatic islet, 18 pancreases were obtained from pancreatectomy at Asan Medical Centers in Seoul, Korea. The IRB of Asan Medical Centers approved this study (2013-06-19) and all subjects underwent an informed consent process. Non-tumor pancreatic tissues were used for islet isolation. Islet purification was done using the Ricordi method, with a COBE device [21]. The average of age of the study subjects (n = 18) was 55±16 years. The control group consisted of eight males and ten females, including two males who were previously diagnosed with T2D.

### DNA methylation data

The DNA methylation data set was produced by the Infinium Human Methylation 450K Beadarray platform, which interrogates >485,000 CpG dinucleotides. The DNA methylation procedure was described in detail in a previous paper [6]. GenomeStudio V2011 (Methylation Module, R 2.11) software was used for quantification and image analysis of the methylation data (Illumina, San Diego, CA, USA). All samples passed the GenomeStudio quality control steps based on built in control probes for staining, hybridization, extension and specificity, and the bisulfite conversion efficiency was high (intensity signal >4000) [22]. Each methylation data point was identified by fluorescent signals from the M (methylated) and U (unmethylated) alleles. Background intensity, computed from a set of negative controls, was subtracted from each analytical data point. The ratio of the fluorescent signals from the two alleles was then computed as ß = (max(M, 0))/(|U| + |M| + 100). The ß-value reflects the methylation level at each CpG site. A ß-value of 0–1.0 was reported to signify percent methylation, from 0% to 100%, respectively, for each CpG site.

The average of beta data of a total of 52 samples corrected for background signal was generated from a chip experiment. Subjects were divided into two subgroups (ΔhiGlu60 subgroup: cases/controls: 8/8; type 2 diabetes subgroup: cases/controls: 5/5) with paired samples at the 1st and 5th stages. We assessed the difference in methylation profiles between cases and controls for each comparison group. We identified the significant 250 CpG sites filtered by the difference between the average methylation values of the cases and the controls (significance was set at p values <0.05).

Statistical significance of the methylation data was determined using the paired t-test, in which the null hypothesis was that no difference exists between the means of groups in the methylation data. R scripts were used for all other analytical processes.

Whole DNA methylation data set of 52 blood samples is available (the accession number 2011–06) under the approval of data access committee of the National Biobank of Korea (http://www.nih.go.kr/NIH/eng/contents/NihEngContentView.jsp?cid=65714&menuIds=HOME004-MNU2210-MNU2327-MNU2329-MNU2338).

### Pyrosequencing

Pyrosequencing assays were designed, optimized, and performed on the PSQ HS 96A System (Biotage AB) according to the manufacturer’s specifications (Pyrosequencing, Qiagen, USA). cg23586172 was not available by pyrosequencing, and we determined the DNA methylation of chr17:55484635, which is closest to cg23586172 (chr17:55484600). chr17:55484635 is named by chromosome base pair location (hg19). A PCR set for pyrosequencing was performed for the CpG site, chr17:55484635 (*MSI2*). The primer sequences for MSI2 were as followed: the forward (5'-biotin-AGGGGAAGAAAAAAA GAAAATAAGAG-3'), the reverse (5'-AACTCTCCTCA CACATACAATATCAA-3') and the sequencing primer (5'-CACCTACACAAAAAACCC-3'). The sequence for analyze is CRAAACTAAAAAATCRCAAA. The PCR amplification was done for 37 cycles with an annealing temperature of 61°C.

### Whole genome bisulfite sequence (WGBS) library construction & sequencing

The sequencing libraries were prepared with KAPA DNA Library Preparation Kits (Kapa Biosystems, KK8201) according to the manufacturer’s instructions. Briefly, fragmentation of 5 μg of genomic DNA was performed using adaptive focused acoustic technology (AFA; Covaris).

DNA was converted with the EpiTect Bisulfite Kit (Qiagen, 59104) according to the manufacturers' instructions. The bisulfite-converted DNA libraries were PCR-amplified with 4 PCR cycles using PfuTurbo Cx DNA polymerase (Agilent, 600410). The final purified product was then quantified using qPCR according to the qPCR Quantification Protocol Guide and qualified using the Agilent Technologies 2100 Bioanalyzer (Agilent). Then, sequences were determined using the HiSeq platform (Illumina).

### WGBS data processing and methylation profile calling

The quality of paired end sequencing reads (100 bp) generated from WGBS was verified with FastQC (version 0.10.0). Trimmomatic (version 0.32) was used to remove adapter sequences and bases with base quality lower than 3 from the end reads. The minimum phred quality score was set to 30 and the minimum read length was half of the original read length. The cleaned reads were aligned to the *homo sapiens* genome (UCSC hg19) using BSMAP based on the SOAP (Short Oligo Alignment Program) [23]. BSMAP (version 2.87 parameter set -v 2 -r 0) allowed up to 2 nucleotide mismatches to the reference genome per seed and returned only uniquely mapped reads. Mapped data (SAM file format) were sorted and indexed using SAMtools (version 0.1.19)[24]. Afterwards, PCR duplicates were removed with Picard Mark Duplicates (version 1.11) (https://broadinstitute.github.io/picard/).

Methylation level was assessed with the BSMAP program [23]. The methylation ratio of every cytosine with a CT count greater than 10 was considered a reliable methylation call. For regions covered by both ends of a read pair, only one read was used to call methylation. The resulting coverage profiles are summarized as # of C / effective CT count for each of the three sequence contexts (CG, CHG, and CHH). In the human genome, which has about 28 million CpGs, at least 1 billion 100 bp end reads are needed to get approximately 30X average coverage for WGBS. WGBS data from healthy islets is available via the IHEC data portal (http://epigenomesportal.ca/ihec/) under accession numbers (IHECRE00001871.1, IHECRE00001865.1, IHECRE00001870.1, IHECRE00001862.1, IHECRE00001863.1). The raw WGBS of 5 islet data has been deposited in the EGA database under accession number (EGAS-00001001774).

### Statistics

The DNA methylation data set (Infinium Beadarray) was used for the discovery of DMPs in peripheral blood based on the KoGES. The details of the data collection process are explained well by Shim et al. [6]. Statistical significance of the methylation data was determined using paired t-tests, in which the null hypothesis was that no difference exists between the mean of groups in the methylation data.

For the pyrosequencing studies, Welch’s two-sample t-test was used to calculate *P* values. For the DNA methylation replication study using pyrosequencing, age, sex, BMI, WBC, and RBC were used as the covariates for linear regression analysis. Correlation coefficients between DNA methylation and other traits were calculated with Pearson’s test and graphed. R scripts were used for linear regression coefficient calculation, other analytical and graphic processing (http://www.r-project.org/). To analyze the WGBS data on pancreatic islets, the association between controls and cases was analyzed with Mann-Whitney U test using the R program.

## Results

### DNA methylation in relation to hyperglycemia

We aimed to identify a differential methylation profile of peripheral blood related to hyperglycemia. The discovery study compared blood DNA methylation of 13 control and 13 cases subjects. Table 1 summarizes the categories of two cases and control groups for the discovery set. For our 10-year longitudinal analysis, differential DNA methylation was first identified in 52 blood samples (1st and 5th, two time points from 26 samples).

For the discovery set (total n = 26), we classified the subjects into two subgroups by OGTT results: ΔhiGlu60 (cases/controls: 8/8) and T2D (cases/controls: 5/5).

We compared the peripheral blood methylation status (beta value) of 26 subjects in the 1st and 5th stages for longitudinal analysis by paired t-test. To identify specific sites of differential DNA methylation associated with the effect of age in each subgroup (at the 1st and 5th stages), we performed a paired *t*-test for differential methylation. Information on the filtering conditions and detailed differentially methylated positions (DMPs) is given in Table 2. We selected the 250 top-ranking stage 5-specific DMPs from the ΔhiGlu60 and T2D subgroups (p values <0.05 and top 250 higher delta mean values). The stage 5-specific DMP data are shown for both the controls and the cases of the ΔhiGlu60 subgroup and the T2D subgroup (S1 Table).

**Table 2:** Summary of the DMP criteria for the discovery study.

Subgroup		Stage 5th -specific DMP	Filtering condition	Case- Specific DMP
**ΔhiGlu60**	**Controls**	250	Ranking by the methylation difference between cases and controls (nominal p values <0.05)	
	**Cases**	250	Ranking by the methylation difference between cases and controls (nominal p values <0.05)	229
**type 2 diabetes**	**Controls**	250	Ranking by the methylation difference between cases and controls (nominal p values <0.05)	
	**Cases**	250	Ranking by the methylation difference between cases and controls (nominal p values <0.05)	153

DMP = differential methylation CpG position p values

The Illumina ID list and DMP data for stage 5th -specific DMPs from the four subgroups (control and cases in the three subgroups) are documented with averages of DNA methylation along with *P* values in S1 Table. After longitudinal selection, we analyzed the case-specific DMPs for the two groups. Finally, DMPs were selected by case-control analysis. We identified a total of 382 DMPs (153 T2D-specific DMPs and 229 ΔhiGlu60-specific DMPs) by subtraction of case-specific DMPs from control DMPs in each subgroup. The case-specific DMPs are marked in bold in S1 Table. The gene and the methylation information are also documented in S1 Table. Three hundred and eighty-two DMPs were discovered and mapped to 280 annotated genes (S1 Table).

**Fig 1:**
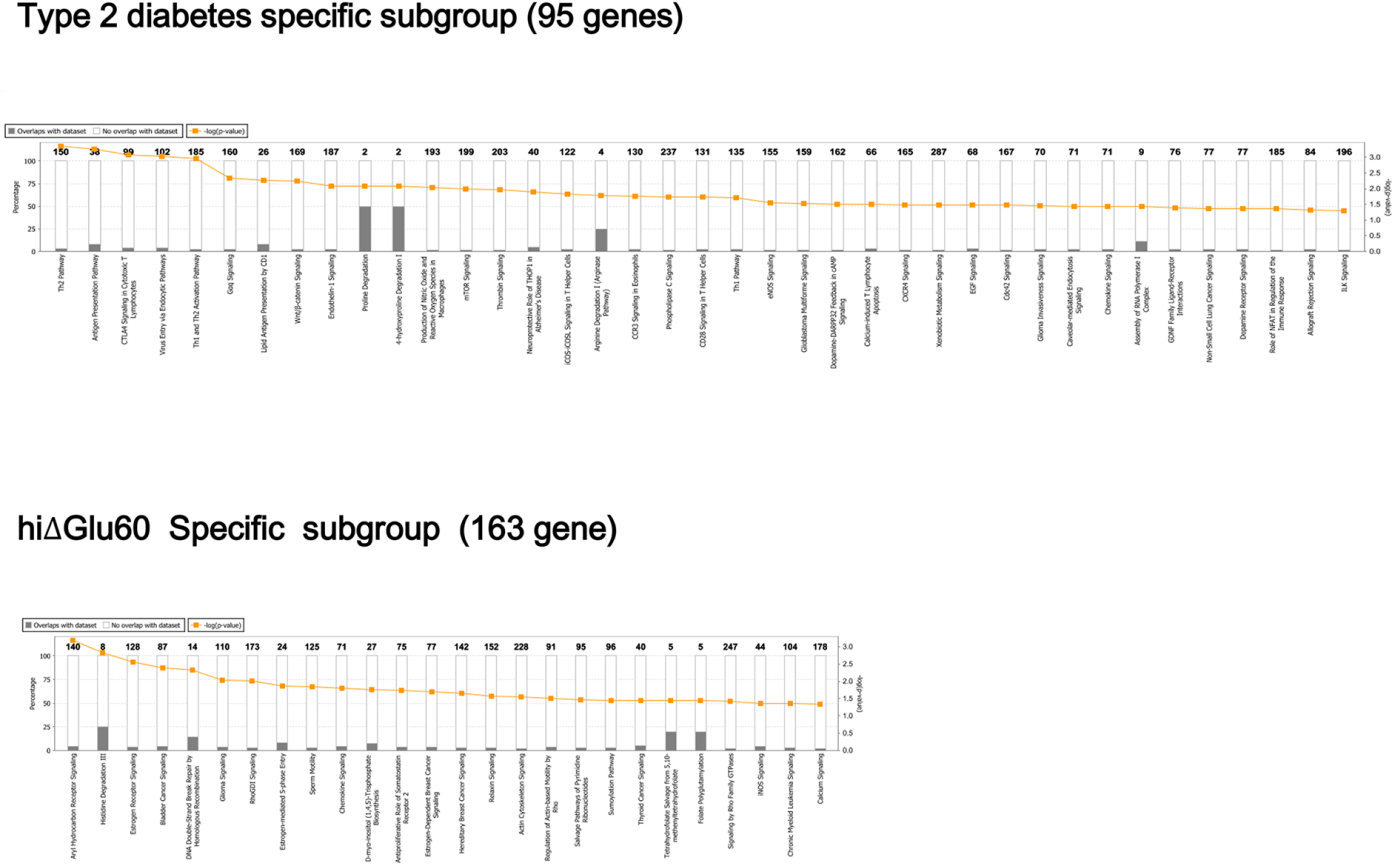
Canonical pathways enriched in the T2D and ΔhiGlu60 subgroups by Ingenuity Pathway Analysis (IPA). The bar graphs showed 39 pathways in the T2D and 26 pathways in the ΔhiGlu60 subgroups with the enriched percentage and p values. The percentages indicate the number of differentially methylated genes that map to each pathway divided by the total number of genes that map to the canonical pathway. The total number of gene was indicated at the top of bar. The p value (–log10 *P*) is the probability that each biological function assigned to that data set was assigned by chance.

We presents enriched biological term and biological function analyses of the differentially methylated gene sets of the T2D (100 genes) and ΔhiGlu60 (180 genes) subgroups, respectively. The biological functions associated with these gene sets were evaluated using Ingenuity Pathways Analysis (IPA, Ingenuity Systems, www.ingenuity.com). T2D-specific (95 genes) and ΔhiGlu-specific (163 genes) symbols were recognized by the software and entered into the enrichment analyses (Fig 1). The left axis on each graph, the percentages, indicates the number of differentially methylated genes that map to the pathway divided by the total number of genes that map to the canonical pathway. The right axis, the p value (–log10 P), is the probability that each biological function assigned to that data set was assigned by chance.

The biological roles of the differentially genes were examined using a range of analyses. The results showed that differentially methylated genes in 39 group gene ontology biological processes were over-represented in the T2D subgroup, and 26 were over-represented in the ΔhiGlu60 subgroup.

The top IPA canonical pathways that were enriched in the T2D subgroup were associated with the Th2 (T helper) pathway. The others were associated with immune signaling, and were involved in such processes as the antigen presenting pathway, CTLA4 (Cytotoxic T-Lymphocyte Associated Protein 4) signaling in cytotoxic T lymphocytes, and viral entry via endocytosis. In the ΔhiGlu60 subgroup, the top three canonical pathways were associated with aryl hydrocarbon receptor signaling, histidine degradation III, and estrogen receptor signaling.

We investigated the possible overlap between the DMPs in the subgroups, under the assumption that overlapping DMPs may provide more significant and stronger candidates related to consistent epigenetic changes that occur in response to glucose homeostasis. Three DMPs (cg22604213, cg23586172, and cg25290098) in the T2D subgroup overlapped with those in the ΔhiGlu60 subgroup (S1 Fig). Two DMPs mapped to the *MSI2* and *CXXC4 (CXXC Finger Protein 4)* genes (except cg25290098). The two DMPs were both hypo-methylated (S1 Table). The DMP in *MSI2* was hypo-methylated by 11% in T2D cases (p value = 0.0038) and 7% in ΔhiGlu60 cases (p value = 0.038). The DMP in *CXXC4* was also hypo-methylated by 15% in T2D cases (p value = 0.044) and 12.8% in ΔhiGlu60 cases (p value = 0.033).

### Replication analysis of target DMP in blood via pyrosequencing

We further examined replicate target DMPs in an expanded T2D group in the KoGES dataset (total n = 440). Table 3 summarizes the categories within the T2D group (Glu0 > 126 mg/dL) for the replication study. The controls (n = 220) were age- and sex-matched with the subjects in the T2D group (n = 220). The inclusion criteria are described in the Experimental Procedures section. We investigated three DMPs in the replication study by pyrosequencing.

**Table 3:** Summary of the subjects for the target CpG methylations by pyrosequencing in the replication study.

	Controls	Cases	p values
**n** (male/female)	220 (118/102)	220 (118/102)	
**Age** (yr)	61.14±9.34	60.12±7.99	0.2015
**BMI** (kg/m^2^)	23.79±3.05	25.63±2.98	**< 0.0001**
**HbA1c** (%)	5.43±0.46	7.62±1.29	**< 0.0001**
**Glu0** (mg/dL)	92.23±7.60	165.51±54.30	**< 0.0001**
**Ins0** (*u*U/*ml*)	8.34±3.36	16.71±22.00	**< 0.0001**
**HOMA-IR**	1.91±0.81	7.49±12.83	**< 0.0001**
***QUICKI***	0.3522±0.02	0.3060±0.030	**< 0.0001**
**WBC** (10^3^/*u*L)	1.313±2.02	1.317±2.02	0.7969
**RBC** (10^6^/*u*L)	5.370±1.50	5.347±1.52	**< 0.0001**
**chr17:55484635** (*MSI2*)	29.46±3,69	26.35±3.33	**< 2.20E-16**

The values are indicated by mean±SD, nominal p values: Welch’s two-sample t-tests were performed.

Target pyrosequencing of DMPs combines a simple reaction protocol with reproducible and accurate measures of degree of methylation [25, 26]. The PCR reactions designed for *CXXC4* failed, so the CpG site for *MSI2* was available for pyrosequencing. The details of *MSI2* target DMP analyzed via pyrosequencing are described in Materials and Methods. Pyrogram for *MSI2* target sites is shown in S2 Fig. In Table 3, pyrosequencing analysis showed that *MSI2* had significant associations with T2D (n = 440, p value =: 2.20E-16). The effect on glucose homeostasis of the identified blood DMP was in the same direction, hypo-methylated. In particular, the degree of CpG methylation was on average decreased by 3% at chr17:55484635 (*MSI2*).

### Target DMPs associated with other diabetic traits

We discovered DMPs significantly related to the hyperglycemia group. Furthermore, we found our results to be reproducible. To investigate the potential biological significance of the DMPs, we examined their association with diabetes-related traits (Glu0, HbA1c, BMI, Ins0, HOMA-IR and *QUICKI*) in the T2D group (n = 440). To investigate their potential biological significance, the linear regression coefficients with age, sex, BMI, WBC, and RBC as covariates were determined between each DMP and six traits (Table 4).

**Table 4:** Linear regression of target CpG methylations with six diabetes-related traits.

	Glu0	HbA1C	BMI	Ins0	HOMA-IR	*QUICKI*
	r	r	r	r	r	r
	p value	p value	p value	p value	p value	p value
**chr17:55484635(*MSI2*)**	-4.094	-0.0920	-0.0376	-0.313	-0.2346	2.389E-03
	**9.02E-13**	**3.61E-13**	**5.96E-04**	**2.6E-08**	**1.18E-07**	**2.2E-16**

r: Linear regression coefficient with age, sex, BMI, WBC, and RBC as covariates

*MSI2* had negative associations with Glu0, HbA1c, BMI, Ins0 and HOMA-IR (p values = 9.02E-13, 3.61E-13, 5.96E-04, 2.6E-08 and 1.177E-07, respectively). *MSI2* showed the strongest positive correlation with insulin sensitivity, *QUICKI* (r = 0.0023 p value = 2.2E-16).

**Fig 2:**
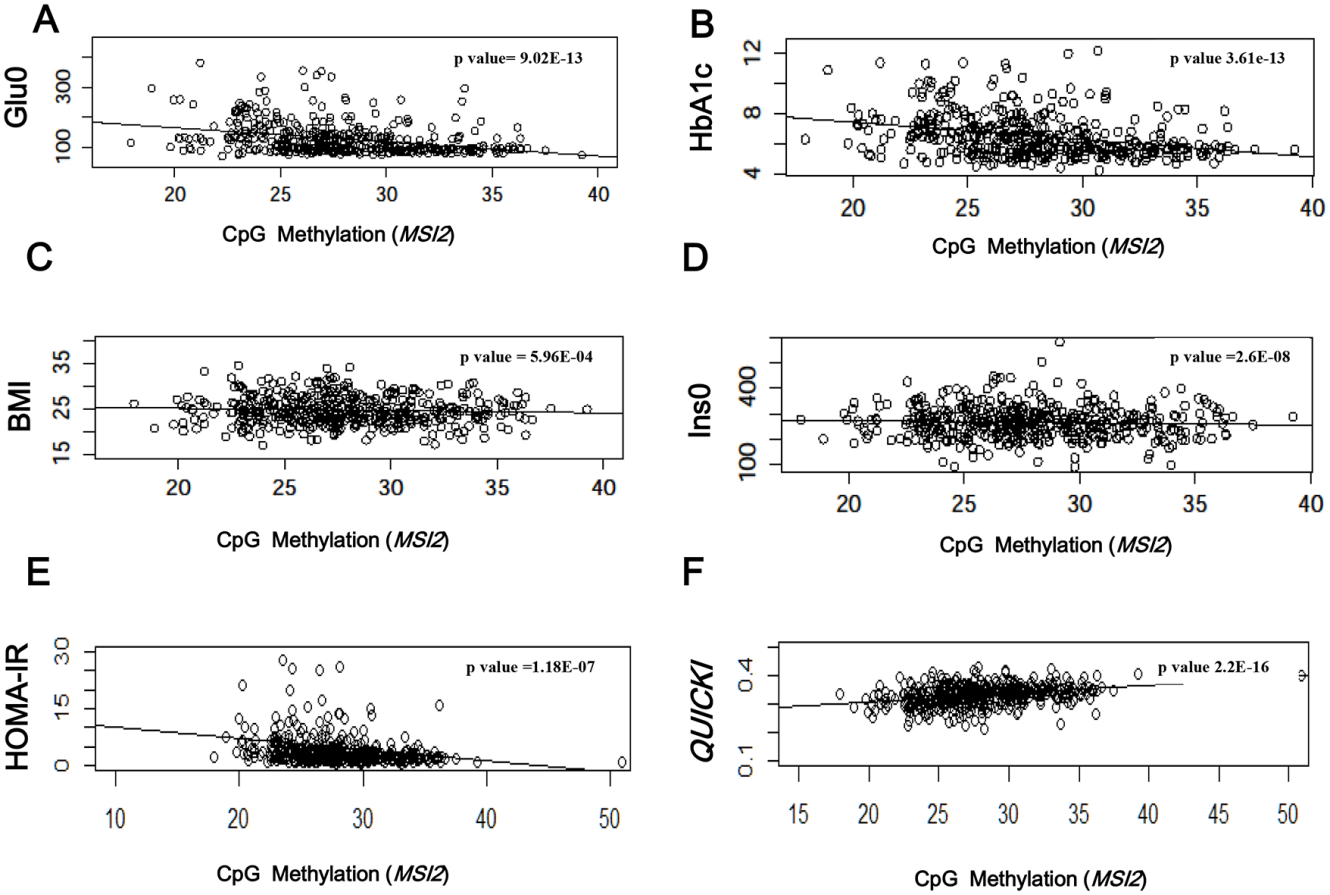
Correlations of the degree of DNA methylation of *MSI2* (chr17:55484635) with six related traits. Correlations between DNA methylation at cg23586172 and Glu0 (A), HbA1c (B), BMI (C), Ins0 (D), HOMA-IR (E) and *QUICKI* (F) were present in the T2D group (n = 440). Glu0, fasting blood glucose level; HbA1, glycosylated hemoglobin level; BMI, body mass index; and Ins0, fasting blood insulin level. HOMA-IR, Ins0(μU/mL) × Glu0 (mg/mL)/405): *QUICKI*, 1 / (log(Ins0 μU/mL) + log(Glu0 mg/dL).

The DMP correlation graphs (chr17:55484635) between *MSI2* and the six traits are shown with beta values (except HbA1c) in Fig 2. The correlation graph clearly shows that the glycemic traits (Glu0 and HbA1c) decreased (Fig 2A and 2B) and insulin sensitivity (*QUICKI*) increased with an increase in DNA methylation (Fig 2F). There were negative correlations between BMI or fasting insulin level and DNA methylation in graphs (Fig 2C and 2D), and beta value was -0.0376 for BMI and -0.313 for Ins0. Insulin resistance (HOMA-IR) and insulin sensitivity (*QUICKI*) were inversely related with DNA methylation (Fig 2E and 2F).

### Investigation of target DMPs from eighteen WGBS maps of human islets

The differential CpG methylation sites in blood cells (*MSI2*) were strongly associated with T2D. Their annotated gene expression in blood was associated with the postprandial high glycemic group. Specifically, DMPs were significantly correlated with glucose traits. Prolonged high blood glucose levels can affect the epigenetic signature of target cells in all organs simultaneously. The pancreas consistently senses and controls blood glucose level.

Pancreatic islets consist of five cell types that secrete hormones directly into the blood to maintain glucose homeostasis [27]. Life-long high blood glucose concentrations may affect pancreatic cells and change the epigenetic structure of the pancreas. T2D patients who lose glycemic control may have different epigenetic signatures in their pancreatic islets.

We investigated DNA methylation of the *MSI2* genes in pancreatic islets to identify differential methylation associated with T2D. We integrated 18 deep-coverage (30X) nucleotide-resolution whole-genome methylation maps of human islets. Single base resolution methylome maps of islet DNA isolated from donors were produced using whole genome bisulfite sequence (WGBS). Eighteen islets were purified from the pancreas after surgery from two T2D and sixteen healthy subjects, all of whom gave informed consent. The details of the WGBS method and patient information are described in the Methods section.

**Fig 3:**
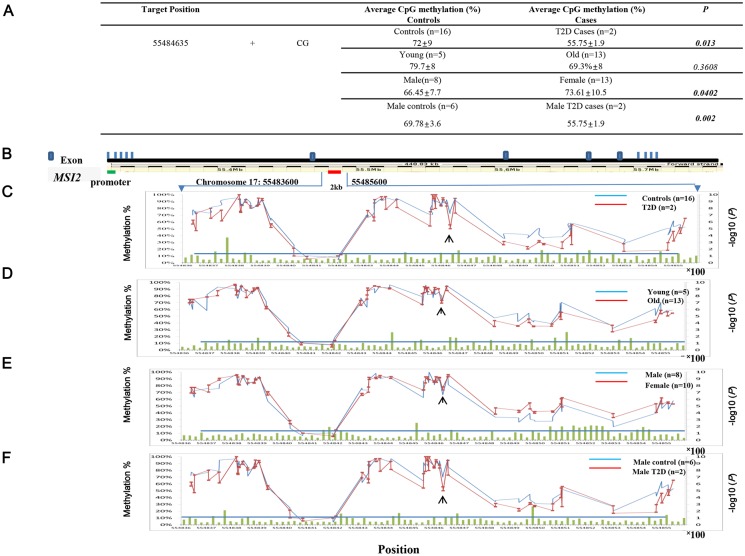
DNA methylation mapping of *MSI2* in human pancreatic islets. A. Estimate of the average methylation of chr17:55484635 (chromosome 17:55484635) and the complimentary CpG site (chromosome 17:55484636) in controls (n = 16) and T2D subjects (n = 2). The p values are given. B. Schematic *MSI2* gene structure was described with 13 exons. DNA methylation map of the 2 kb region (chromosome 17:55483600 to 55485600) centered on the target site (chr17:55484635). C. The average normal methylation values (n = 16) from chr17:55483600 to chr17:55485600 are graphed in blue and the average T2D methylation values (n = 2) are in red. The—log10 (*P*) of 86 DMPs is indicated by the green bar on the bottom of the methylation curve. The line at -log10 (p value = 0.05) shows the cutoff for statistical significance. The arrow indicates the position at chromosome 17:55484635. D. Differential methylation map drawn after classifying 18 islets into cases and controls by age (“young” < 40 yrs and “old” > 40 yrs). The average methylation values of young islets (n = 5) are graphed as controls in blue, and those of old islets (n = 13) are shown as cases in red. E. Differential methylation map drawn after classifying 18 islets into cases and controls by gender (“male” and “female”). The average methylation values of male islets (n = 8) are graphed in blue, and those of female islets (n = 10) are shown in red. F. Differential methylation map drawn after classifying 8 male islets into T2D cases and controls after sex matching. The male T2D cases (n = 2) are shown in red, and the male controls (n = 6) are in blue.

chr17:55484635 methylation in *MSI2* was dramatically decreased in T2D subjects (Fig 3A). The average chr17:55484635 methylation (genomic position ch17:55484635, hg19) was 72% in normal islets and 56% in T2D islets (Δ16%, p value = 0.013). The average methylation values and p values of chr17:55484635 after classifying 18 islets into cases and controls by age (“young” < 40 yrs and “old” > 40 yrs) and gender (“male” and “female”) are also shown in Fig 3A. DMPs were significantly more hyper-methylated in the female group than in the male group (p values <0.05). DMPs in the old islet group were more often hypo-methylated than in the young group, but the difference was not significant (p values >0.05). Males with T2D exhibited significantly more hypo-methylation than did male controls after sex matching (p values <0.05).

Differential methylation has been linked to numerous phenotypes, including age and gender. Two studies showed that beta cell number did not differ given gender and T2D status [14, 28]. Gene-specific sex differences in DNA methylation are associated with altered expression and insulin secretion in human islets [28]. Therefore, we analyzed differential methylation after classifying 18 islets into cases and controls by age (“young” < 40 yrs and “old” > 40 yrs) and gender (“male” and “female”).

The average methylation values of young islets (n = 5) are graphed as controls in blue, and those of old islets (n = 13) are shown as cases in red (Fig 3D). Percentage-wise, old islets were hypo-methylated along the 2 kb region. To show our analysis by gender, the average methylation values of male islets (n = 8) are graphed in blue, and those of female islets (n = 10) are shown in red (Fig 3E). Because the effect of gender is important, we compared T2D cases and controls after sex matching. The male T2D cases (n = 2) are shown in red, and the male controls (n = 6) are shown in blue (Fig 3F). The differential CpG methylation at chr17:55484635 was significant even after sex matching. Our data included a small number of T2D cases (n = 2), however, the case-control study classifying the data by age and sex suggested that differential DNA methylation of chr17:55484635 was specific to T2D. There were similar patterns of differential methylation among the age, T2D, and sex-matched T2D graphs, but not the gender graphs, along the 2 kb DMR (differentially methylated region).

We expanded our search to a ±5 kb (10 kb) region centered at the target site (chromosome 17: 55484635) to analyze differential DNA methylation changes. There were 292 DMPs in that 10 kb region (S2 Table). Information about the 292 DMPs with average methylation values in normal and T2D subjects are described, along with *P*-values, in S2 Table. The methylation of 171 CpG sites decreased in T2D subjects, and the average decrease at 59 CpG sites was >10%. Thirty-nine statistically significant DMPs (sDMPs) were found in T2D pancreatic islets (p values < 0.05). Thirty-six of those 39 sDMPs were hypo-methylated. The average hypo-methylation change was 13.9% (Table 5). When classified by age (5 young, < 40 yrs and 13 old, > 40 yrs), we found 39 sDMPs in the ±5kb (10kb) region surrounding the target site (chr17:55484635) of *MSI2* in older patients, but the average methylation change was only 0.1%. The effects of age on DMR were random. When classified by gender (8 males and 10 females), there were 30 sDMPs in females with an average 9.5% methylation change. The sex effect was in a similar direction (26 of 30 sDMPs), mostly hyper-methylated, in females. We classified T2D islet group by sex to check the gender effect for the differential methylation of *MSI2* DMR (Table 5). The T2D cases were all males, so we only used males as controls (n = 6). The average hypo-methylation change of 32 sDMPs was 11.3%, compared to 13.9% without taking gender into account. The DMRs near chr17:55484635 in pancreatic islets were generally significantly hypo-methylated in T2D cases.

**Table 5:** Number and extent of methylation change of DMPs by cases and controls in target DMRs in 10 kb region from 18 WGBS of human islets.

Classification	The numbers of controls	The numbers of cases	IncreasedDMPs(n)	Decreased DMPs(n)	sDMP(p values < 0.05)	Average methylation changes(%) of sDMP(CI)
**T2D**	16	2	171	121	39	13.91%(4.18 ~ 17.06%)
**Age**	5 (young)	13 (old)	150	142	39	0.12%(-11.17 ~ 3.77%)
**Sex**	8 (males)	10 (females)	129	163	30	-9.57%(-16.76 ~ -6.89)
**T2D(Sex-matched)**	6 (males)	2 (males)	174	117	32	11.31%(-2.50 ~ 16.37)

sDMP = significant differential methylation CpG position, CI = confidence interval

p values: independent two sample t-test assuming unequal variance (Mann-Whitney U test) was performed.

We investigated CpG methylation in the promoter region of the *MSI2* gene at chromosome 17 55332163: 55334373, which may be important for gene expression in islets. The *MSI2* gene showed a high degree of enrichment in CpG sites (280 sites) in the 2 Kb promoter regions, including the plus and minus strands. Most CpG sites in the promoter region were extremely hypo-methylated, and there was zero methylation at 225 CpG sites. None of the 280 CpG sites were significantly associated with T2D (p values >0.05).

## Discussion

This study revealed significant changes in DNA methylation associated with hyperglycemia. Extended exposure to high blood glucose levels can affect the epigenomic signatures of target cells in all organs simultaneously. We identified the DMPs in blood from two small subgroups by longitudinal and cross-sectional analyses. In the replication study, the *MSI2* methylation change was significant and had strong association with glycemic traits.

We found the same association in blood and pancreatic islet DNA in subjects with T2D, implying that *MSI2* methylation is biologically relevant. Although some reports of whole blood and islet methylation profiles are available, they do not relate methylation indices to T2D [13, 15]. The epigenetic perturbation of *MSI2* in T2D was much greater in islet cells compared to circulating blood (Δ16% versus Δ3%).

The hypo-methylation of a target CpG site in islets was associated with T2D, but differential methylation may not relate to *MSI2* expression, because it is located in intron 6. There were no significant DNA methylation changes in the promoter regions of *MSI2* in islet cells; all were hypo-methylated. By searching the UCSC genome browser (http://genome.ucsc.edu), we found that chr17:55484635 was located in the enhancer regions of several cell lines, including hematopoietic blood, embryonic stem, liver, cancer, breast, and skin cells. Therefore, differential methylation of chr17:55484635 may affect regulation of other genes [29, 30].

Other questions arise, such as whether these DMPs are the result of changes in blood and islet cell heterogeneity. In blood cells in the replication study, there was no significant difference in WBC counts between the control and case groups (Table 3). We also used WBC and RBC as covariates for regression analysis. However, blood contains many different immune cell types, and changes may depend on long-term hyperglycemia. Blood cell heterogeneity could affect CpG methylation, and can depend on particular CpG sites. Therefore, we analyzed the linear regression of DMP in *MSI2* with Glu0 and Ins0 in the T2D case group (n = 220). The correlations were in the same direction (r = -2.6092 and r = -0.6496, respectively), and were significant for Ins0 (p value = 8.077E-08). For islet cell heterogeneity, Dayeh and Ling et al. found no significant differences in beta cell content and purity in islets of non-diabetic subjects compared with T2D human donors [14].

We also investigated the effect of DNA methylation by age and sex in islets (Table 5). Aging did not affect average DNA methylation in the ±5kb (10kb) region surrounding the target site (chr17:55484635) of *MSI2*, but sex did. Finally, whether sex-matched or not, hypo-methylation of *MSI2* was T2D-specific in our results (Table 5).

Musashi proteins exist as two isoforms, *MSI1* and *MSI2*. Their expression is known to be related to hematopoetic stem cell activity and myeloid leukemia [31–33]. Szabat et al. showed that lipotoxicity and ER stress could upregulate *MSI2* via a non-canonical pathway and suggested a modulatory role for this pathway in type 2 diabetes [34]. They demonstrated that *MSI2* overexpression in mouse pancreatic beta cell line (MIN6) significantly decreased *Ins1* (insulin 1) and *Ins2* (insulin II) gene expression, whereas *Msi2* knockdown increased *Ins1* and *Ins2* expression.

Unfortunately, we did not investigate gene expression of *MSI2* in islet cells especially with T2D, but the DMP (chr17:55484635) of *MSI2* may not directly relate to its RNA expression. However, differential methylation of *MSI2* (chr17:55484635) was associated with insulin sensitivity (*QUICKI*, p value = 2.2E-16) and resistance (HOMA-IR, p value = 1.177E-07). The positive correlation of *MSI2* gene expression in blood with 60 min and 120 min insulin level does not completely support *in vitro MSI2* overexpression experiment which result in decreased *Ins* gene expression, but may explain the sequential relationship of *MSI2* gene expression in response to hyperglycemia. Our findings suggest that epigenetic perturbation on the target site (chr17:55484635) of *MSI2* gene in circulating blood and pancreatic islets should represent or affect hyperglycemia.

## Supporting information

S1 Fig Schematic diagram of differential methylation of CpG sites (DMPs) in the subgroups. A total of 153 T2D-specific DMPs and 229 ΔhiGlu60-specific DMPs are in each circle. Three DMPs (cg22604213, cg23586172, cg25290098) were common to the T2D and ΔhiGlu60 subgroups. Three DMPs are indicated by overlapping circles with annotated gene names (*CXXC4* and *MSI2*; excepting cg25290098).
(PPTX)

S2 Fig The pyrogram for *MSI2*. Grey area with CpG number indicates the DMP (chr17:55484635) that was analyzed.
(PPTX)

S1 Table Information of Top 250—5th stage specific DMPs of control T2D, case T2D, control ΔhiGlu60 and case ΔhiGlu60 by longitudinal analysis. (XLSX)

S2 Table The DNA methylations of two hundred and ninety two DMPs measured a ±5 kb (10 kb) region centered at the target site (chromosome 17: 55484635) between the normals and T2D islets. (XLSX)
