# Supplementary material for: Differential DNA methylation of MSI2 and its correlation with diabetic traits
Source: PLoS One. 2017 May 24;12(5):e0177406. doi: 10.1371/journal.pone.0177406 (PMC5443489; doi:10.1371/journal.pone.0177406)
Supplement: S1 Fig — A total of 153 T2D-specific DMPs and 229 ΔhiGlu60-specific DMPs are in each circle. Three DMPs (cg22604213, cg23586172, cg25290098) were common to the T2D and ΔhiGlu60 subgroups. Three DMPs are indicated by overlapping circles with annotated gene names (CXXC4 and MSI2; excepting cg25290098). (PPTX) [file pone.0177406.s001.pptx]

## Slide 1
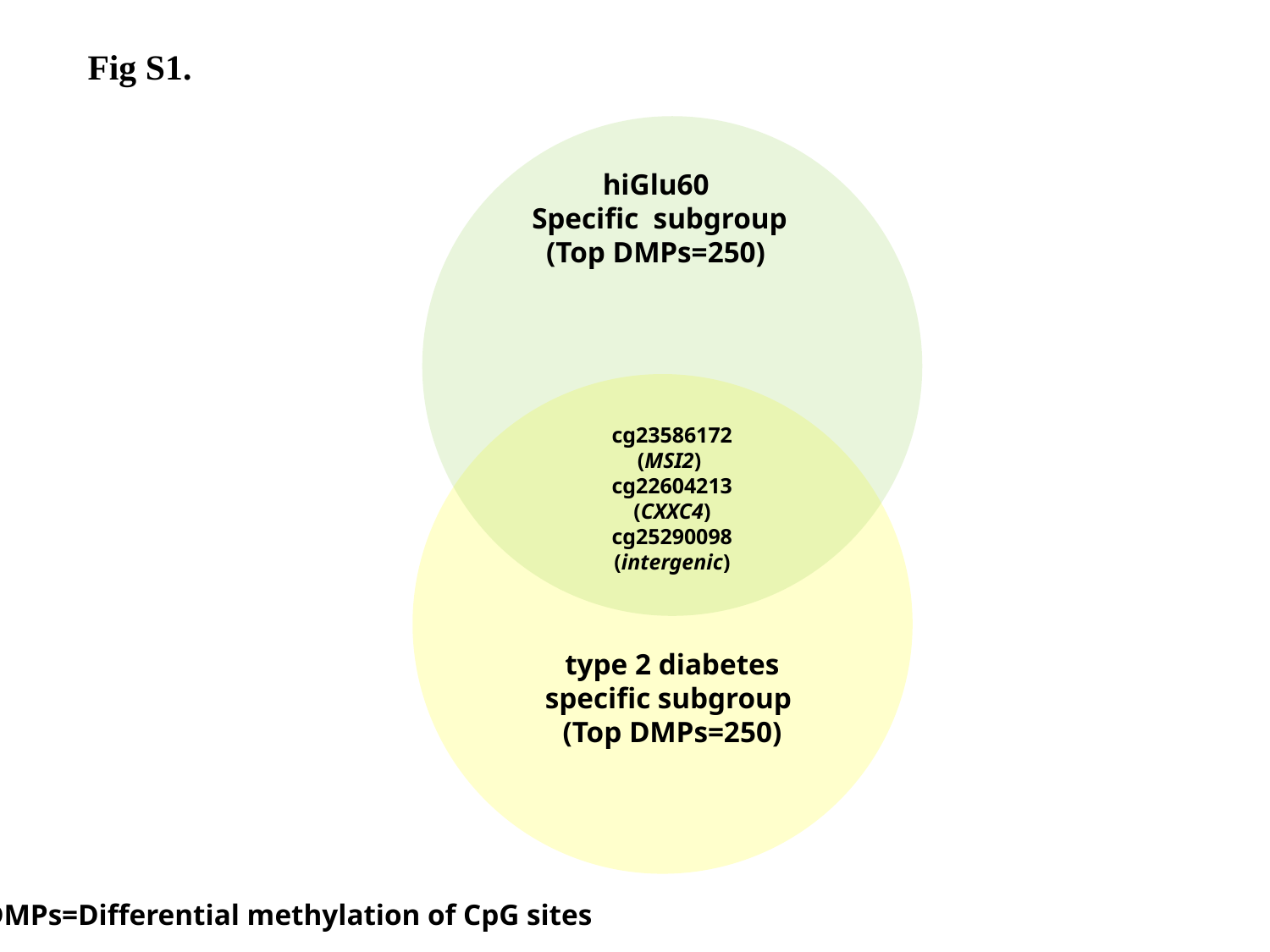

Fig S1.
cg23586172
(MSI2)
cg22604213
(CXXC4) cg25290098
(intergenic)
type 2 diabetes specific subgroup
(Top DMPs=250)
DMPs=Differential methylation of CpG sites
