# Supplementary figures and images for: Differential DNA methylation of MSI2 and its correlation with diabetic traits
Source: PLoS One. 2017 May 24;12(5):e0177406. doi: 10.1371/journal.pone.0177406 (PMC5443489; doi:10.1371/journal.pone.0177406)

## Slide 1
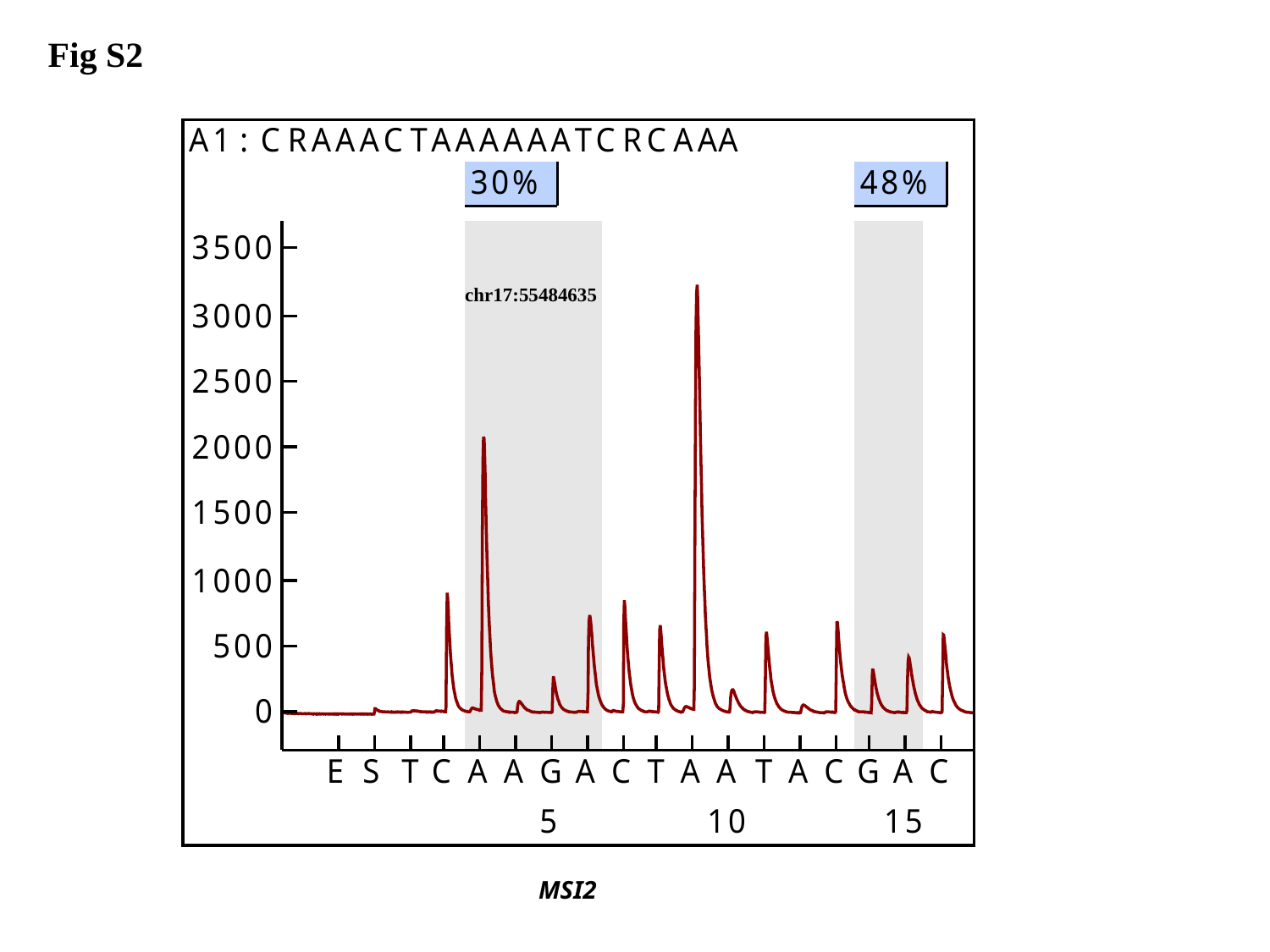

Fig S2
chr17:55484635
MSI2

Supplement: S2 Fig — Grey area with CpG number indicates the DMP (chr17:55484635) that was analyzed. (PPTX) [file pone.0177406.s002.pptx]
